# An alphanumeric classification of osteoporotic pelvic ring injuries

**DOI:** 10.1007/s00402-020-03546-9

**Published:** 2020-07-31

**Authors:** Dietmar Krappinger, Verena Kaser, Anke Merkel, Sabrina Neururer, Richard A. Lindtner

**Affiliations:** 1 Department of Trauma Surgery, Medical University of Innsbruck, Anichstraße 35, 6020 Innsbruck, Austria; 2 Department of Radiology, Medical University of Innsbruck, Innsbruck, Austria; 3 Department of Medical Statistics, Informatics and Health Economics, Medical University of Innsbruck, Innsbruck, Austria

**Keywords:** Pelvic ring injury, Pelvic ring fracture, Pelvic fracture, Osteoporotic fracture, Fragility fracture, Insufficiency fracture, Pelvis, Osteoporosis, Classification, Reliability, Low-energy, FFP classification

## Abstract

**Introduction:**

Classification and management of osteoporotic pelvic ring injuries (OPRI) continue to pose a considerable challenge to orthopaedic traumatologists. The currently used fragility fractures of the pelvis (FFP) classification of OPRI has recently been shown to have significant weaknesses. The aim of this study therefore was to propose a new, simple, yet comprehensive alphanumeric classification (ANC) of OPRI and to assess its intra- and interobserver reliability. Furthermore, its potential advantages over the FFP classification are discussed.

**Materials and methods:**

One hundred consecutive CT scans from patients with OPRI were evaluated by three orthopaedic traumatologists with varying levels of experience and one musculoskeletal radiologist. Intra- and interobserver reliability of the proposed classification system was assessed using weighted kappa (*κ*) statistics and percentage agreement. In addition, the Fleiss’ kappa statistic was computed to assess interobserver agreement among all four raters.

**Results:**

Overall intraobserver reliability of the proposed ANC was substantial [*κ* ranging from 0.71 to 0.80; percentage agreement: 70% (range, 67–76%)]. Overall interobserver reliability between pairs of raters was substantial as well [*κ* ranging from 0.61 to 0.68; percentage agreement: 58% (range, 53–61%)]. For ANC types, groups and subgroups, intra- and interobserver reliability were substantial to almost perfect. Interobserver agreement among all four raters was moderate to substantial, with Fleiss’ kappa values of 0.48, 0.69, 0.71 and 0.52 for ANC overall, types, groups and subgroups, respectively.

**Conclusion:**

The proposed ANC of OPRI demonstrated overall reliability comparable to that of the FFP classification. The ANC, however, is simple, more comprehensive, and consistently relates to injury severity.

## Introduction

Osteoporotic pelvic ring injuries (OPRI) are associated with significant morbidity and mortality and their incidence has dramatically increased over the past decades [1–7]. OPRI encompass a wide spectrum of injuries ranging from isolated nondisplaced unilateral pubic rami fractures to combined anterior and posterior pelvic ring injuries involving a displaced H-type sacral fracture with resulting spinopelvic instability. Treatment options range from nonoperative treatment to triangular lumbopelvic stabilisation. However, high-quality studies on optimal treatment of specific OPRI patterns are still lacking. Current treatment recommendations are either expert opinions [8, 9] or based on retrospective studies with small sample sizes [10, 11].

There is general consensus that low-energy OPRI differ in various aspects from high-energy pelvic ring injuries in younger patients. Traditional classification systems, such as the Tile/AO or Young and Burgess classification, were primarily developed for high-energy injuries and are thus of limited value for grading OPRI [8, 12, 13]. At present, there is only one comprehensive classification system specifically developed for OPRI and thus taking account of their specific characteristics: the fragility fractures of the pelvis (FFP) classification proposed by Rommens and Hofmann in 2013 [8]. Although this classification represents an important first step towards a more thorough grading of OPRI for clinical and research purposes, a recent study identified significant weaknesses of this system [14].

The aim of this paper therefore was (1) to propose a new alphanumeric classification (ANC) of OPRI and (2) to evaluate its intra- and interobserver reliability. Additionally, the proposed ANC and the FFP classification were compared in terms of comprehensiveness, simplicity, reliability and relation to injury severity and treatment.

## Materials and methods

### The classification system

The proposed alphanumeric classification (ANC) of OPRI is summarised in Table 1. It is based on injury localisation and morphology. It applies a scheme similar to the widely used alphanumeric AO/OTA fracture classification system and should therefore be familiar to and immediately applicable for orthopaedic trauma surgeons. The classification system differentiates between injury types (A, P and AP), groups (1–3) and subgroups (1–3). The degree of instability increases from group A to group AP and from (sub-)group 1 to (sub-)group 3.

**Table 1:** The alphanumeric classification (ANC) of osteoporotic pelvic ring injuries (OPRI)

Types		Groups^a^		Subgroups^a^	
A	Isolated anterior OPRI	1	Unilateral posterior OPRI	1	Incomplete sacral fracture
P	Isolated posterior OPRI	2	Bilateral posterior OPRI	2	Complete sacral fracture
AP	Combined anterior and posterior OPRI	3	Bilateral posterior OPRI crossing the sagittal midline (i.e. U-, H- or Y-sacral fracture variants)	3	Extrasacral posterior OPRI^b^

^a^Only type P and AP injuries are further divided into groups and subgroups

^b^Extrasacral posterior OPRI include SI-joint disruptions, crescent fracture dislocations and iliac fractures resulting in transiliac instability

#### Types

The types describe the involvement of the anterior and/or posterior pelvic ring. Isolated fractures of the anterior pelvic ring are the most stable and benign OPRI and are therefore classified as type A. Isolated injuries of the posterior pelvic ring are classified as type P, while combined injuries of the anterior and posterior pelvic ring are classified as type AP.

#### Groups

Groups describe the uni- or bilateral involvement of the posterior pelvic ring. Accordingly, type A injuries (isolated fractures of the anterior pelvic ring) are not further subdivided into groups and subgroups. Unilateral posterior pelvic ring injuries are classified as group 1, while bilateral posterior pelvic ring injuries are classified as group 2. Group 3 injuries include bilateral posterior pelvic ring injuries crossing the sagittal midline (i.e. bilateral vertical sacral fractures with a transverse fracture component (also known as U-, H- and Y-type sacral fractures) as well as other less common multiplanar sacral fracture variants resulting in spinopelvic instability).

#### Subgroups

**Fig. 1:**
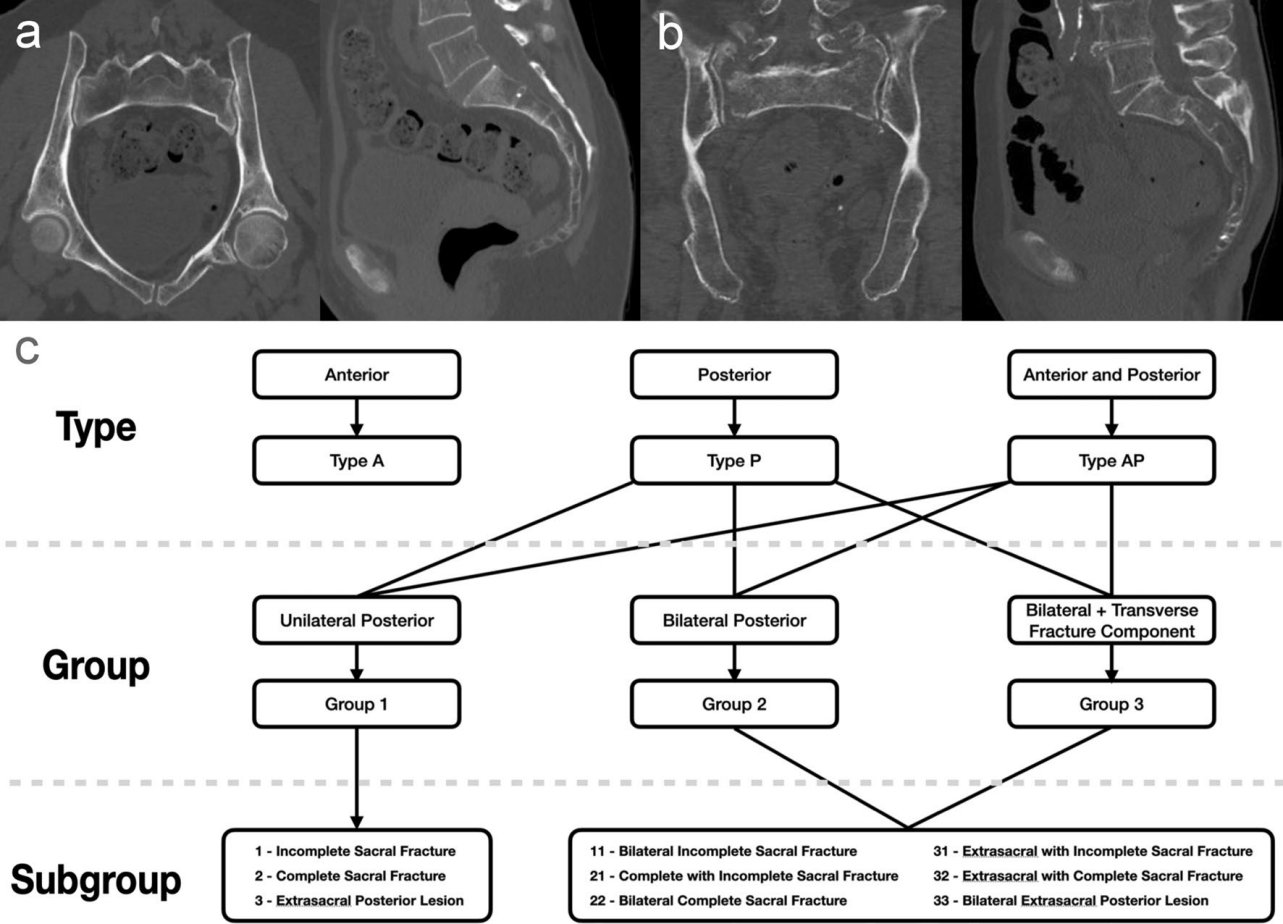
**a** Pelvic CT scan of a 79-year old female after a simple fall. Fracture of the anterior and posterior pelvic ring (type AP). The sacral fracture is unilateral without a transverse fracture component crossing the sagittal midline (group 1). The sacral ala fracture is incomplete involving the anterior cortex only (subgroup 1). This osteoporotic pelvic ring injury (OPRI) is therefore classified as AP1.1. This was the most common OPRI pattern in our study. **b** Pelvic CT scan of an 86-year old female six weeks after a simple fall. Fracture of the posterior pelvic ring only (type P). The sacral fracture is bilateral with a transverse fracture component crossing the sagittal midline (group 3). The sacral ala fracture is bilaterally complete involving both the anterior and posterior cortex (subgroup 2). This OPRI is therefore classified as P3.22. **c** Flowchart diagram of the ANC: types (A/P/AP), groups (1/2/3) and subgroups (1/2/3; single-digit number to specify unilateral and double-digit number to specify bilateral posterior pelvic ring injury patterns)

Subgroups describe the anatomic location of the posterior pelvic ring injuries and the extent of sacral alar fractures. The vast majority of posterior pelvic ring injuries in OPRI occur in the sacral ala. It is therefore reasonable to further divide sacral fractures into two subgroups: Incomplete sacral fractures involving the anterior (or the posterior) cortex of the sacrum only are classified as subgroup 1, while complete sacral fractures involving both the anterior and posterior cortex are classified as subgroup 2. In contrast, extrasacral disruptions of the posterior pelvic ring, such as SI-joint disruptions, crescent fracture dislocations and iliac fractures resulting in transiliac instability, are relatively rare in elderly individuals who sustained a low energy trauma. These typically highly unstable injuries are summarised in subgroup 3. For bilateral injuries of the posterior pelvic ring a double-digit number with the higher number first is used for the subgroup classification. For example, a bilateral sacral alar fracture, which is incomplete on one side and complete on the contralateral side, is classified as subgroup 21. Figure 1a, b shows two typical examples of OPRI and the application of the ANC. Figure 1c illustrates the systematics of the ANC.

Accordingly, the ANC is based on the degree of instability. Instability increases from type A to type AP and from (sub-)group 1 to (sub-)group 3. For example, OPRI with involvement of the anterior and posterior pelvic ring (type AP) are more unstable than those with involvement of the anterior ring only (type A). Bilateral sacral fractures are more unstable than unilateral sacral fractures and bilateral sacral fractures with a transverse fracture component (i.e. multiplanar U-, H- and Y-variants) are more unstable than bilateral sacral fractures without a transverse fracture component. Finally, complete sacral fractures are more unstable than incomplete fractures.

### Validity

After Institutional Review Board approval, 100 consecutive CT scans of patients with OPRI were retrospectively reviewed. Inclusion criteria were (1) OPRI after low-energy trauma or without history of trauma and (2) patient age of at least 70 years. Exclusion criteria comprised (1) OPRI after high-energy trauma, (2) age < 70 years, (3) osteolytic pelvic lesions and (4) metal implants in the pelvic ring.

Three orthopaedic traumatologists with varying levels of clinical experience (a second-year resident, a first-year consultant and an experienced senior pelvic surgeon) and a musculoskeletal radiology consultant independently reviewed the CT scans. The raters were instructed to analyse the injury pattern in each case and to classify the injury pattern according to both the proposed alphanumeric classification system (ANC, Table 1) and the FFP classification system [14] at the same time to allow for direct comparison of reliability values. The axial pelvic CT dataset as well as standard coronal and sagittal two-dimensional reconstructions were available to the raters in a PACS system. Furthermore, a multiplanar reconstruction tool (IMPAX EE, Agfa Healthcare) allowed for two-dimensional reconstruction of the pelvic ring in any arbitrary plane. In a second session 2 months after the first session, the case order was scrambled and each of the four independent raters repeated the classification process to assess intraobserver reliability. For the description of the patients’ collective, such as the relative distribution of fracture patterns, the classification by the most experienced senior pelvic surgeon was used as a reference.

### Statistical analyses

IBM SPSS v24.0 software (Chicago, IL, USA) was used for all analyses. Inter- and intraobserver reliability were assessed by calculating percentage agreement as well as linear-weighted kappa coefficients (*κ*). For the assessment of interobserver reliability, data from both sessions were used. Owing to the fact that only ANC type P and AP (but not type A) injuries are further divided into groups and subgroups, group and subgroup ratings were missing in those cases classified as ANC type A injuries. As recommended by De Raadt et al. [15], the missing ratings were dealt with by applying the pairwise deletion method (i.e. complete-case analysis) for calculation of ANC group and subgroup agreement. In addition to the weighted kappa statistics (interobserver agreement between pairs of raters), the Fleiss’ kappa statistic was computed to assess interobserver agreement among all four raters. Interpretation of the kappa values was performed according to the guidelines of Landis and Koch [16], with kappa values of 0.01–0.20 indicating slight agreement; 0.21–0.40, fair agreement; 0.41–0.60, moderate agreement; 0.61–0.80, substantial agreement; and > 0.80 (almost) perfect agreement.

## Results

A total of 100 consecutive CT scans from 86 females and 12 males with OPRI (mean age: 83.6 years, range 70.1–97.4 years) were included in our study. Two females had undergone a CT scan on the day of trauma as well as an additional CT scan due to persisting pain after 42 and 77 days, respectively. Of these 100 CT scans, 79 had been performed within the first two weeks after trauma, 14 later than 14 days after trauma and 7 in absence of history of trauma.

In 79 of 100 CT scans, at least one posterior pelvic ring lesion was detected, while no posterior lesion was found in 21 CT scans (type A). In total, there were 101 posterior pelvic ring lesions (57 unilateral and 22 bilateral) and 93 of these involved the sacral ala.

**Fig. 2:**
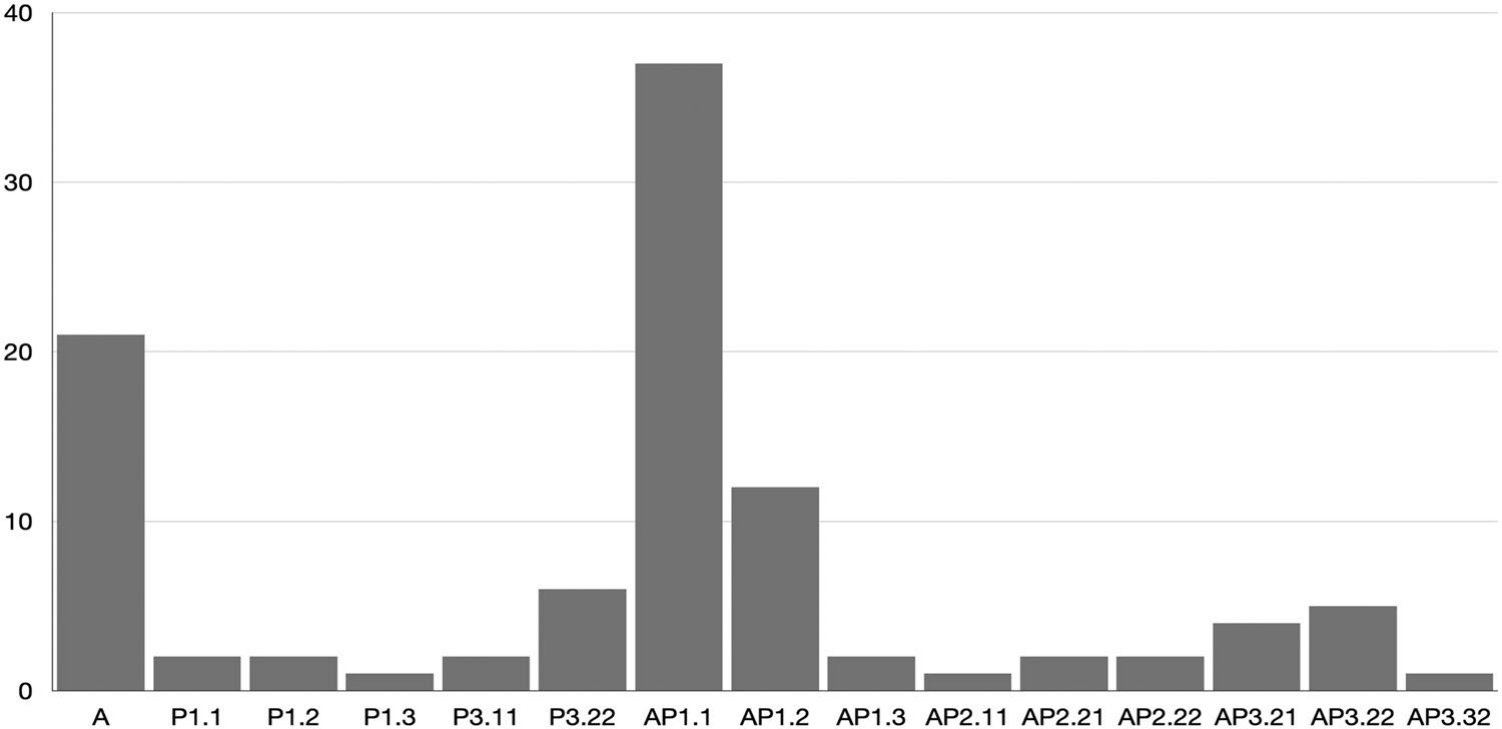
Relative distribution of OPRI patterns (in percent) according to the alphanumeric classification (ANC) (as classified by the pelvic surgeon; *n* = 100 CT scans)

The most frequent OPRI patterns according to the ANC were AP1.1, A and AP1.2, together accounting for 70% of all OPRI (Fig. 2). These patterns were typically observed and diagnosed at an early stage after low-energy falls, with 90% (63/70) of the respective CT scans performed within two weeks after trauma. P3.22, AP3.22 and AP3.21 were the next most common OPRI patterns. In these, 67% (10/15) of the CT scans were performed later than two weeks after trauma or in absence of history of trauma.

The overall intra- and interobserver reliability of the proposed ANC of OPRI are shown in Table 2. Intraobserver agreement was substantial, with linear-weighted kappa coefficients (*κ*) ranging from 0.71 to 0.80 and mean percentage agreement of 70% (range, 67–76%). Overall interobserver agreement between all pairs of raters was substantial as well, with *κ* ranging from 0.61 to 0.68 and mean percentage agreement of 58% (range, 53–61%). None of the 100 cases was deemed unclassifiable by any of the observers.

**Table 2:** Overall inter- and intraobserver reliability of the proposed alphanumeric classification (ANC): percentage agreement and weighted *κ* coefficients (*n* = 100 CT scans; interobserver reliability was assessed using data from both sessions)

	Pelvic surgeon		Consultant		Resident		Radiologist	
Pelvic surgeon	**71%**	**0.75**	61%	0.64	56%	0.64	60%	0.61
Consultant	61%	0.64	**76%**	**0.80**	59%	0.68	56%	0.62
Resident	56%	0.64	59%	0.68	**67%**	**0.79**	53%	0.63
Radiologist	60%	0.61	56%	0.62	53%	0.63	**67%**	**0.71**

Intraobserver reliability values are given in bold

Table 3 shows intra- and interobserver reliability for grading OPRI by ANC types regardless of groups and subgroups (type A/P/AP). There was substantial to almost perfect intra- [*κ* ranging from 0.77 to 0.86; mean agreement: 91% (88–94%)] and substantial interobserver agreement [*κ* ranging from 0.64 to 0.76; mean agreement: 86% (85–90%)].

**Table 3:** Inter- and intraobserver reliability for the assessment of ANC types (A/P/AP): percentage agreement and weighted *κ* coefficients (*n* = 100 CT scans; interobserver reliability was assessed using data from both sessions)

	Pelvic surgeon		Consultant		Resident		Radiologist	
Pelvic surgeon	**90%**	**0.78**	85%	0.64	85%	0.67	85%	0.66
Consultant	85%	0.64	**91%**	**0.78**	90%	0.76	86%	0.70
Resident	85%	0.67	90%	0.76	**94%**	**0.86**	87%	0.70
Radiologist	85%	0.66	86%	0.70	87%	0.70	**88%**	**0.77**

Intraobserver reliability values are given in bold

*ANC* alphanumeric classification

Intra- and interobserver reliability for grading OPRI by ANC groups (group 1/2/3) is shown in Table 4. Intraobserver agreement was almost perfect [*κ* ranging from 0.81 to 0.96; agreement: 91% (88–98%)], while interobserver agreement was substantial to almost perfect [*κ* ranging from 0.73 to 0.86); agreement: 86% (83–92%)].

**Table 4:** Inter- and intraobserver reliability for the assessment of ANC groups (1/2/3): percentage agreement and weighted *κ* coefficients (interobserver reliability was assessed using data from both sessions)

	Pelvic surgeon		Consultant		Resident		Radiologist	
Pelvic surgeon	**89%**	**0.81**	92%	0.86	86%	0.75	87%	0.78
Consultant	92%	0.86	**98%**	**0.96**	85%	0.77	85%	0.74
Resident	86%	0.75	85%	0.77	**88%**	**0.82**	83%	0.73
Radiologist	87%	0.78	85%	0.74	83%	0.73	**90%**	**0.82**

Intraobserver reliability values are given in bold

*ANC* alphanumeric classification

Table 5 shows intra- and interobserver reliability for grading OPRI by ANC subgroups (subgroup 1/2/3). Both intraobserver agreement [*κ* ranging from 0.75 to 0.92; agreement: 75% (69–83%)] and interobserver agreement [*κ* ranging from 0.65 to 0.84; agreement: 65% (56–73%)] were substantial to almost perfect.

**Table 5:** Inter- and intraobserver reliability for the assessment of ANC subgroups (1/2/3): percentage agreement and weighted *κ* coefficients (interobserver reliability was assessed using data from both sessions)

	Pelvic surgeon		Consultant		Resident		Radiologist	
Pelvic surgeon	**74%**	**0.82**	70%	0.84	63%	0.75	73%	0.78
Consultant	70%	0.84	**83%**	**0.92**	67%	0.76	62%	0.73
Resident	63%	0.75	67%	0.76	**69%**	**0.75**	56%	0.65
Radiologist	73%	0.78	62%	0.73	56%	0.65	**74%**	**0.81**

Intraobserver reliability values are given in bold

*ANC* alphanumeric classification

In addition to linear-weighted kappa coefficients (describing interobserver agreement between pairs of raters), Fleiss’ kappa coefficients were calculated to assess interobserver agreement among all four raters. Fleiss’ kappa coefficient values were 0.48 (moderate) for overall interobserver agreement, 0.69 (substantial) for ANC types, 0.71 (substantial) for ANC groups and 0.52 (moderate) for ANC subgroups.

## Discussion

In this paper, we have proposed a new, simple yet comprehensive alphanumeric classification (ANC) of OPRI. Our results indicate that overall intraobserver reliability is substantial and interobserver reliability is moderate (Fleiss’ kappa statistics) to substantial (weighted kappa statistics). Furthermore, the ANC allows to overcome significant shortcomings of the currently used FFP classification, particularly with regard to comprehensiveness, simplicity, reliability and relation to injury severity and treatment:

### Comprehensiveness

The comprehensiveness of a fracture classification implies that all possible fracture patterns are covered by the classification system. Although the FFP classification covers the vast majority of OPRI in 11 subcategories, there are OPRI that are not definitely classifiable according to the FFP classification. This applies, for example, for bilateral incomplete sacral fractures without anterior pelvic ring involvement, because FFP type IIa injuries include isolated unilateral nondisplaced sacral fractures only. Similarly, nondisplaced iliac fractures without anterior pelvic ring involvement are not covered by the FFP classification. Another issue is that the FFP classification neither differentiates between uni- and bilateral posterior pelvic ring injuries in type IIb and IIc OPRI, nor does it specifically consider transverse sacral fracture components at all.

In comparison, comprehensiveness is one of the strengths of the proposed ANC. It covers all OPRI patterns and combinations in a well-known hierarchic system of fracture types, groups and subgroups, including those not classifiable according to the FFP classification. For example, bilateral incomplete sacral fractures without involvement of the anterior pelvic ring are classified as P2.11 or as P3.11 (depending on the presence of a transverse sacral fracture component). Uni- and bilateral injuries of the posterior pelvic ring in all their possible combinations are clearly distinguished depending on injury location, completeness of the sacral fracture and presence of a transverse sacral fracture component crossing the sagittal midline.

### Simplicity

Developing a classification system that is impressively simple, but also comprehensive, and has an acceptable number of (sub)categories, but does not oversimplify may be like squaring the circle. Accordingly, a classification system invariably represents a compromise between simplicity and selectivity. The FFP classification consists of four main fracture types with a total of 11 subtypes. The definition and distinction of the four main fracture types of the FFP classification (I: anterior only; II: nondisplaced posterior lesions; III: displaced unilateral posterior lesions; IV: displaced bilateral posterior lesions) is clear and simple. This is also true for the distinction of subtypes in type I, III and IV injuries. It is, however, less intuitive for type II injuries, which represent the majority of OPRI [8, 14]. Type IIa injuries include unilateral nondisplaced sacral fractures without anterior involvement, but they explicitly exclude iliac fractures. Type IIb and IIc injuries share an additional injury of the anterior pelvic ring. Type IIb injuries on the one hand cover sacral fractures only, while type IIc injuries on the other hand additionally include nondisplaced iliac fractures and iliosacral disruptions. This appears to be quite confusing at first glance and may decrease simplicity of the FFP classification scheme.

In comparison, the ANC of OPRI consists of 31 categories and thus provides substantially higher selectivity than the FFP classification, which includes 11 categories. Nevertheless, simplicity of the ANC is ensured by the strictly hierarchic system of types, groups and subgroups. Therefore, mastering the simple systematics of the ANC already results in knowledge about all 31 classification categories. Additionally, the ANC applies a scheme similar to the widely used AO/OTA fracture classification systems and should therefore be familiar to and immediately applicable for traumatologists.

### Inter- and intraobserver reliability

A recent study evaluated the reliability of the FFP classification and reported overall moderate to substantial interobserver and substantial to almost perfect intraobserver agreement [14]. For the assessment of complete nondisplaced vs. displaced sacral fractures (FFP IIc, IIIc and IVb injuries), however, inter- and intraobserver reliability was markedly lower (unweighted *κ* ranging from 0.10 to 0.52 and from 0.28 to 0.66, respectively; linear-weighted *κ* ranging from 0.17 to 0.64 and from 0.35 to 0.76, respectively) [14]. The latter may be partly due to the relatively vague definition of “displacement” by Rommens and Hofmann (i.e. “deformation of the anatomical landmarks” and “widening and gap formation between fracture fragments”). Another recent study involving 4 raters and 97 OPRI cases found slight to moderate (unweighted *κ* ranging from 0.18 to 0.47) interobserver and fair to moderate (unweighted *κ* ranging from 0.29 to 0.55) intraobserver reliability of the FFP classification [17]. The most recent study by Pieroh et al. [18] applied Fleiss’ kappa statistic and involved 13 raters of varying level of expertise, 3 classification cycles by each rater and 60 OPRI cases. The authors reported moderate intraobserver reliability [mean Fleiss’ kappa coefficient: 0.46 (95% confidence interval: 0.40 to 0.50)] and moderate interobserver reliability [0.53 (0.48–0.58)] for the complete FFP classification. For the FFP main group classification, intraobserver reliability was moderate [0.60 (0.53–0.65)] and interobserver reliability was substantial [0.61 (0.54–0.66)].

In comparison, the ANC showed comparable overall intra- and interobserver reliability although it distinguishes almost three times the number of classification categories. Furthermore, the intra- and interobserver reliability for grading OPRI by ANC types, groups and subgroups was substantial to almost perfect (Tables 3 and 4). Nevertheless, one might argue that the ANC does not distinguish between nondisplaced and displaced sacral fractures. However, the vague definition of “displacement” in the FFP classification resulted in poor reliability and therefore reduced its overall usability. In our opinion, even a more precise criterion for displacement, such as a threshold of 2 or 5 mm, is hardly measurable with adequate reliability in OPRI and would be an arbitrary threshold as well. Second, OPRI result from low-energy trauma or without history of trauma and are therefore typically non- or minimally displaced. Third, the vast majority of fracture classification systems do not specifically include displacement as a parameter, which does not compromise their usability.

### Relation to injury severity

The FFP classification was developed based on the degree of instability. We agree that it is reasonable to assume that OPRI without involvement of the posterior pelvic ring (type I) are more stable than OPRI with posterior pelvic ring involvement (type II–IV), that incomplete sacral ala fractures (type IIb) are more stable than complete fractures (type IIc) and that unilateral posterior pelvic ring injuries (type III) are more stable than bilateral injuries (type IV). However, regarding its relation to injury severity, the main drawback of the FFP classification is that it subsumes OPRI with different degrees of instability into the same FFP type. For example, type IIb and IIc injuries include fractures with uni- or bilateral involvement of the anterior and posterior pelvic ring. Accordingly, these injuries consist of a minimum of two and a maximum of four fractures of the pelvic ring. Moreover, type III injuries are defined as unilateral displaced posterior lesions, but if these injuries are combined with a contralateral nondisplaced posterior lesion, they are still classified as type III. Additionally, type IVc injuries are defined as “combination of different instabilities in the dorsal pelvis” [8]. Finally, transverse sacral fracture components indicating a multiplanar sacral fracture pattern are not considered as a criterion of instability at all.

In comparison, the ANC is based on the degree of instability as well. It clearly differentiates between incomplete and complete sacral fractures, between unilateral and bilateral involvement, and between sacral and extrasacral posterior pelvic ring injuries in all possible combinations. This is enabled by the double-digit subgroup labelling described above. In addition, multiplanar sacral fractures (i.e. H-, U- or Y-type fractures; group 3) with spinopelvic instability are clearly distinguished from sacral fractures without a transverse fracture component (group 1 and 2).

### Relation to prognosis and treatment

A clinically relevant classification system and its categories not only should be related to injury severity but also should be predictive of relevant patient outcomes and aid treatment decision making. Yet, the predictive value of a classification system can be only suspected at the time of proposal, since its assessment requires a large observational study “with accurate recording of most clinically relevant outcomes and their respective known or suspected prognostic factors, including treatment options” (i.e. phase 3 of validation) as pointed out by Audigé et al. [19]. However, in contrast to Rommens et al. whose classification and treatment recommendations are based on injury morphology only [8, 9], we are convinced that a classification system for OPRI should go beyond morphology and include selected clinical and patient-specific factors relevant to prognosis and treatment decision making. The most important of the latters, in our experience, are those summarised in Table 6 and include restrictions in patient mobility as a consequence of sustaining an OPRI, pain and analgesic requirements due to OPRI, preinjury mobility, time trauma to diagnosis of OPRI, and presence of neurological deficits related to OPRI. Nevertheless, future research is needed to identify the most appropriate clinical modifiers to be incorporated into the ANC with the eventual goal of predicting treatment approaches and outcomes in addition to describing OPRI morphology.

**Table 6:** Potential clinical modifiers of the ANC suggested for future evaluation

Modifiers	Specifications
Mobility	Preinjury mobility level is not restricted by OPRI-related pain Preinjury mobility level is restricted by OPRI-related pain (Almost) immobile due to OPRI-related pain
Analgesic requirements	No medication or non-opioid only Opioid for mild to moderate pain Opioid for moderate to severe pain
Preinjury mobility	“Go-go”: unrestricted mobility “Slow-go”: mobile inside house and no (or minimal) mobility outside house “No-go”: bedridden or wheel chair
Time trauma to diagnosis	< 2 weeks > 2 weeks No history of trauma
Neurological status	No neurological deficit related to OPRI Lower extremity radicular symptoms (L5 and/or S1) Bowel and/or bladder dysfunction ± lower extremity radicular symptoms

*ANC* alphanumeric classification, *OPRI* osteoporotic pelvic ring injury

This study has some limitations. First, evaluation of a higher number of cases would have increased the power of our statistical analysis. Second, CT scans but no additional MRI scans were used for reliability assessment. Third, the grading of the 100 OPRI cases by the experienced pelvic surgeon was used as a reference for the description of the patients collective. Fourth, for full validation of the ANC, a pragmatic multicentre agreement study and a prospective clinical observational study have to be conducted in future [19]. Last, the ANC was developed primarily for low-energy OPRI. For the classification of high-energy pelvic ring injuries, the Tile/AO or Young and Burgess classification might be more appropriate even in elderly individuals.

In conclusion, the proposed alphanumeric classification (ANC) of OPRI is a simple, hierarchically organised alphanumeric classification scheme traumatologists may rapidly become familiar with. The ANC provides several advantages over the currently used FFP classification of OPRI, particularly with regard to comprehensiveness, simplicity, and relation to injury severity. Despite the substantially higher number of categories of the ANC, intra- (substantial) and interobserver (moderate to substantial) reliability are comparable to those of the FFP classification. Future research, however, is needed to assess outcomes of different ANC patterns and to clarify indications and most appropriate strategies for operative treatment.
